# Maternal metabolic health drives mesenchymal stem cell metabolism and infant fat mass at birth

**DOI:** 10.1172/jci.insight.146606

**Published:** 2021-07-08

**Authors:** Melissa L. Erickson, Zachary W. Patinkin, Allison M. Duensing, Dana Dabelea, Leanne M. Redman, Kristen E. Boyle

**Affiliations:** 1 Pennington Biomedical Research Center, Louisiana State University, Baton Rouge, Louisiana, USA.; 2 Department of Obstetrics and Gynecology, University of Rochester Medical Center, Rochester, New York, USA.; 3 Department of Pediatrics, University of Colorado Anschutz Medical Campus, Aurora, Colorado, USA.; 4 Department of Epidemiology, Colorado School of Public Health, Aurora, Colorado, USA.; 5 Lifecourse Epidemiology of Adiposity and Diabetes Center, Aurora, Colorado, USA.

**Keywords:** Metabolism, Reproductive Biology, Fatty acid oxidation, Human stem cells, Obesity

## Abstract

Exposure to maternal obesity may promote metabolic dysfunction in offspring. We used infant mesenchymal stem cells (MSCs) to experimentally examine cellular mechanisms of intergenerational health transmission. Our earlier reports show MSCs collected from infants of mothers with obesity had a dichotomous distribution in metabolic efficiency; they were either efficient (Ef-Ob) or inefficient (In-Ob) with respect to fatty acid oxidation (FAO). Here, we sought to determine if this was due to a primary defect in FAO. Accordingly, we measured FAO in myogenic differentiating MSCs under 3 conditions: (a) myogenesis alone, (b) excess fatty acid exposure, and (c) excess fatty acid exposure plus a chemical uncoupler to increase metabolic rate. Compared with normal weight and Ef-Ob MSCs, In-Ob displayed lower FAO in myogenesis alone and after fatty acid plus uncoupler, indicating In-Ob were less metabolically flexible after increasing lipid availability and metabolic rate, demonstrating a primary deficit in FAO. MSC FAO was negatively associated with fasting maternal glucose and insulin and positively associated with fasting HDL-cholesterol. MSC FAO was negatively associated with infant fat mass. These data indicate a less favorable maternal metabolic milieu, independent of maternal BMI, reduces intrinsic MSC FAO and is linked to higher infant adiposity as early as birth.

## Graphical abstract

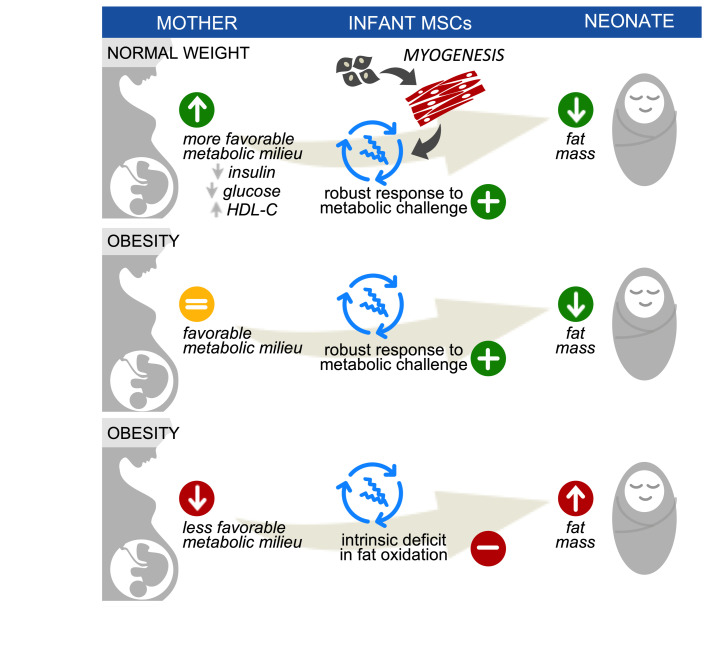

## Introduction

Childhood obesity is a global public health crisis in need of efficacious prevention strategies (1). Several observational studies in humans show that exposure to maternal obesity in utero is associated with obesity and metabolic disease risk later in life (2–10). This is in alignment with the Developmental Origins of Health and Disease (DOHaD) hypothesis, which posits that the propensity for obesity may be affected during fetal development. The DOHaD hypothesis provokes the notion that intervening to improve maternal health will in turn improve offspring health, which illuminates maternal metabolic health endpoints as novel clinical treatment targets. Efforts to empirically test the DOHaD hypothesis at the cellular and molecular levels are limited, however, due to ethical issues associated with accessing fetal tissue. Previous research by ourselves and others shows the innovative Wharton’s jelly–derived mesenchymal stem cell (MSC) model may overcome this limitation (11–13).

MSCs are derived from umbilical cords collected at birth and can be experimentally differentiated along mesodermal lineages. Mesodermal tissues (e.g., adipose, skeletal muscle) are important for the regulation of whole-body metabolism; thus, the MSC model affords an opportunity to directly interrogate human neonatal samples. These in vitro experiments can be used to probe MSC metabolic function, which may reveal a molecular source of metabolic deficiencies potentially promotive of offspring metabolic disease later in life. We have previously employed this model to examine the effects of maternal obesity on MSC metabolism in cells undergoing myogenesis. In support of the DOHaD hypothesis, our work has shown that maternal obesity alters MSC metabolism by promoting excessive fat storage, dysregulated AMPK, and reduced total mitochondrial fatty acid oxidation (FAO) (11). Moreover, we uncovered 2 distinct phenotypes among MSCs from infants of mothers with obesity: metabolically efficient and metabolically inefficient with respect to FAO (14).

In our previous study (11), metabolic efficiency was assessed as a ratio of acid-soluble metabolites (i.e., partially oxidized fats) to fatty acids completely oxidized to CO_2_ (ASM/CO_2_). A lower ASM/CO_2_ ratio indicates increased metabolic efficiency, whereas a higher ratio indicates lower metabolic efficiency. The previously observed dichotomy in MSC efficiency may be clinically meaningful, as comparisons of maternal metabolic circulating factors revealed corresponding differences in insulin, Homeostatic Model Assessment of Insulin Resistance (HOMA-IR), and free fatty acids. These data suggest that maternal metabolic health may be paramount to transmitting poor metabolic phenotype to offspring, rather than maternal weight status per se. This unanticipated finding supports the importance of deepening our understanding of the MSC metabolic phenotypes. Here, we tested the hypothesis that differences in metabolism among the obese (Ob) MSCs were due to intrinsic deficits in mitochondrial FAO.

Using metabolic flexibility assessments in vitro, we can more clearly define the origin of metabolic inefficiency. Metabolic flexibility is broadly defined as the ability to respond or adapt to conditional changes in metabolic demand (15). Changes in substrate availability or energetic demand are 2 common ways to assess such metabolic flexibility in cells, as we and others have considered previously in myotubes from patients with insulin resistance or type 2 diabetes (14, 16–19). For example, it is possible that inefficient MSCs oxidize fewer fatty acids because there is less fatty acid available for oxidation in the mitochondria, or perhaps basal metabolic rate is lower, leading to a backup of tricarboxylic acid (TCA) cycle products causing fewer fatty acids to be completely oxidized to CO_2_. If neither of these challenges ameliorate differences in metabolism, then lower FAO represents a true deficit in lipid flux within the mitochondria. Therefore, the primary objective here was to compare MSC FAO in response to multiple metabolic challenges, including excess fatty acid exposure and chemical uncoupling, which increases cellular metabolic rate, across 3 MSC metabolic phenotypes (normal weight, efficient-obese, and inefficient-obese). Given that in utero exposures may be driving MSC metabolism, secondary objectives were to examine relationships between MSC fat metabolism and maternal cardiometabolic risk factors during pregnancy, as well as body composition during childhood.

## Results

### MSC donor characteristics

#### Experimental groups.

Examination of MSCs collected from infants of mothers with obesity revealed 2 distinct phenotypes, characterized as either high or low metabolic efficiency (11). The ratio of incomplete to complete fat metabolism (ASM/CO_2_) was used to experimentally characterize MSC metabolic efficiency, as shown in Supplemental Figure 1; supplemental material available online with this article; https://doi.org/10.1172/jci.insight.146606DS1; reproduced with permission from *Molecular Metabolism* (11). Thus, we defined 3 phenotypically distinct groups: MSCs from infants of mothers with normal weight (NW-MSCs), metabolically efficient MSCs from infants of mothers with obesity (Ef-Ob), and metabolically inefficient MSCs from infants of mothers with obesity (In-Ob). Importantly, these 3 groups exhibited a stepwise increase in maternal insulin (11).

#### Maternal.

Participant characteristics for the 3 groups of MSCs including NW-MSCs (*n* = 15), Ef-Ob MSCs (*n* = 5), and In-Ob MSCs (*n* = 9) are shown in Table 1. In earlier reports, we observed significant linear associations between groups using a trend analysis; thus, results in Table 1 consistently report trend analysis results (14). Pre-pregnancy weight (*P* = 8.4 × 10^9^) and BMI (*P* = 2.3 × 10^12^) showed a linear increase across the 3 groups (NW, Ef-Ob, and In-Ob, respectively). Maternal metabolic panel assessed at late gestation also revealed group differences, including a linear decrease in fasting total cholesterol (*P* = 0.027) and HDL-cholesterol (*P* = 0.013) across the 3 groups (NW, Ef-Ob, and In-Ob, respectively). In addition, HOMA-IR (*P* = 0.007) increased linearly across the 3 groups (NW, Ef-Ob, and In-Ob, respectively).

#### Infant.

Participant characteristics of offspring assessed approximately at birth are shown in Table 1. Cord blood insulin increased (*P* = 0.001) across NW, Ef-Ob, and In-Ob groups. Body composition was assessed 24–48 hours after delivery. Percentage fat-free mass decreased (*P* = 0.02) across NW, Ef-Ob, and In-Ob groups. Fat mass, expressed as absolute kilograms (*P* = 0.02) and percentage (*P* = 0.02), increased across NW, Ef-Ob, and In-Ob groups.

### Experimental design

Given our previous observation of divergence among MSCs from infants born to mothers with obesity, suggesting metabolically efficient and inefficient subgroups (11), the primary objective of this study was to determine whether deficits in In-Ob were due to limitations in mitochondrial fat availability or lower cellular metabolic rate that may display as reduced fat metabolism. We measured fat metabolism in myogenic differentiating MSCs under 3 metabolic conditions: (a) myogenesis alone (control, CT), (b) following 24 hours of excess fatty acid exposure (oleate/palmitate mix, 24hFA), and (c) following 24hFA, with fat metabolism measures in the presence of carbonyl cyanide *p*-trifluoromethoxyphenylhydrazone (FCCP), a chemical uncoupler (24hFA+FCCP). If the In-Ob MSCs are less metabolically flexible with respect to complete FAO under the metabolic challenge conditions, this would support our hypothesis that they are truly metabolically inefficient with respect to fat oxidation rather than simply oxidizing available substrate at a lower metabolic rate, as would be evident if the metabolic challenges eliminated differences in fat oxidation.

### Cellular fat content is higher in both Ob MSCs

**Figure 1:**
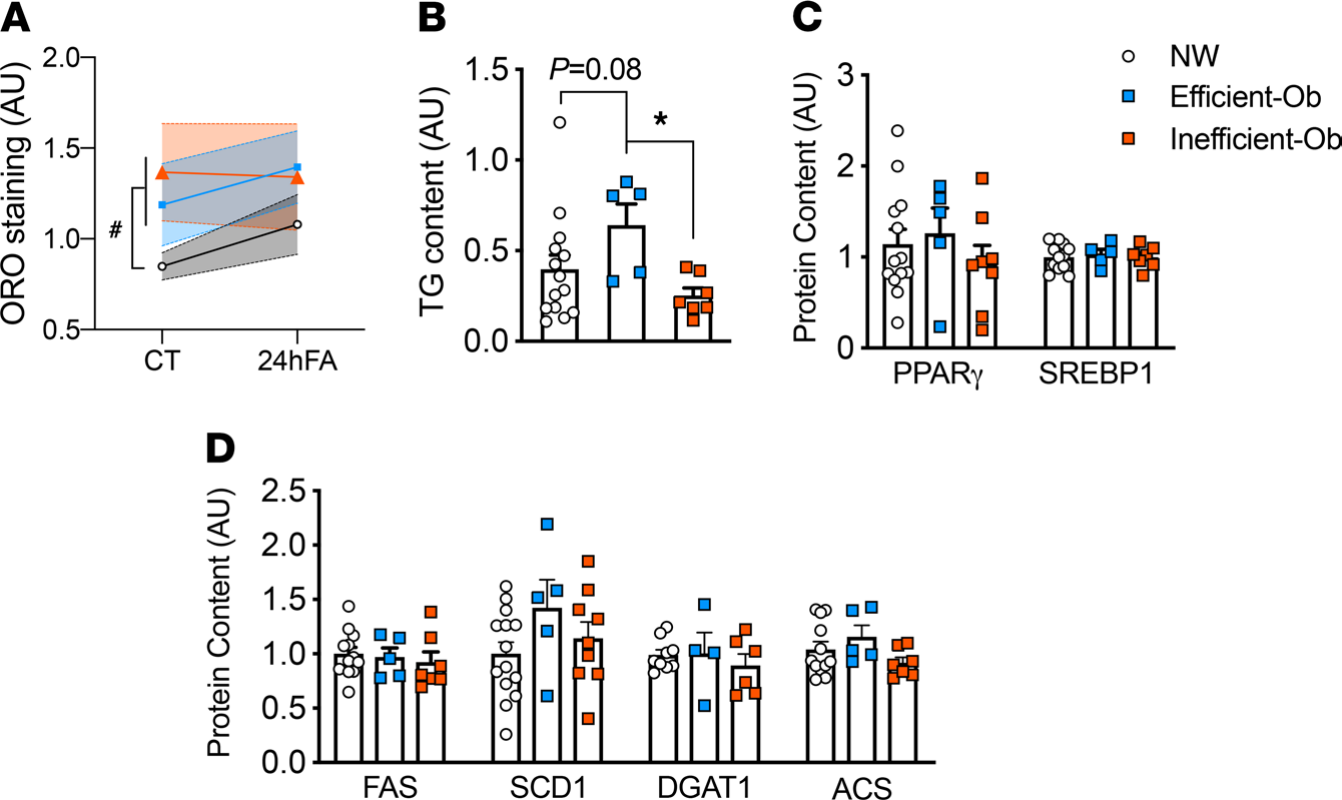
Cellular fat content is higher in both Ob MSCs. (**A**) Cellular lipid content assessed by ORO staining in CT and 24hFA conditions. ORO was higher in both Ef-Ob and In-Ob, compared with NW-MSCs (*t* = 2.25, *P* = 0.04, NW *n* = 13, Ef-Ob *n* = 5, In-Ob *n* = 9) during myogenesis, although this did not persist during 24hFA as assessed by 2-way ANOVA. (**B**) Stored triglyceride (TG) content and phosphorylation of protein involved in lipogenesis, in which Ef-Ob MSCs stored more TGs than In-Ob MSCs (*t* = –2.60, *P* = 0.02, NW *n* = 14, Ef-Ob *n* = 5, In-Ob *n* = 7) as assessed by 1-way ANOVA. (**C**) Protein content of lipogenesis regulators PPARγ or SREBP1 was not different among MSC metabolic phenotypes as assessed by 1-way ANOVA. PPARy: NW *n* = 13; Ef-Ob *n* = 5, In-Ob *n* = 8; SREBP1: NW *n* = 15; Ef-Ob *n* = 5, In-Ob *n* = 7. (**D**) Protein content of lipogenesis proteins FAS, SCD1, DGAT1, and ACS was not different among MSC metabolic phenotypes as assessed by 1-way ANOVA. FAS: NW *n* = 12; Ef-Ob *n* = 5, In-Ob *n* = 7; SCD1: NW *n* = 14; Ef-Ob *n* = 5, In-Ob *n* = 9;DGAT: NW *n* = 9; Ef-Ob *n* = 4, In-Ob *n* = 6; ACS: NW *n* = 12; Ef-Ob *n* = 5, In-Ob *n* = 7. Wes (ProteinSimple) chemiluminescence plots for all proteins are shown in Supplemental Figure 2. ^#^different from both Ob groups; *In-Ob different from Ef-Ob.

We have previously reported that cellular lipid content was higher in Ob MSCs (12). Here, we measured cellular fat content by Oil Red O (ORO) staining in CT and 24hFA conditions. As previously shown, ORO was higher in both Ef-Ob and In-Ob, compared with NW-MSCs (Figure 1A; *t* = 2.24, *P* = 0.04) during myogenesis (CT), but this effect was diminished with 24hFA. To explore this further, we next measured stored triglyceride (TG) content and phosphorylation of proteins involved in lipogenesis. We observed Ef-Ob MSCs stored more TG than In-Ob MSCs (Figure 1B; *t* = –2.60, *P* = 0.02). However, we observed no differences in protein content of lipogenesis regulators peroxisome proliferator-activated receptor γ (PPARγ) or sterol regulatory element binding protein 1 (SREBP1) (Figure 1C), or in lipogenesis proteins fatty acid synthase (FAS), stearoyl-CoA desaturase 1 (SCD1), diacylglycerol acyltransferase 1 (DGAT1), and acetyl CoA synthetase (ACS) (Figure 1D, chemiluminescence plots are in Supplemental Figure 2). Based on these results, it does not appear that enhanced lipogenesis is accounting for differences in cellular fat content observed with ORO staining or TG measures.

### Fat uptake and esterification are not different between MSC metabolic phenotypes

**Figure 2:**
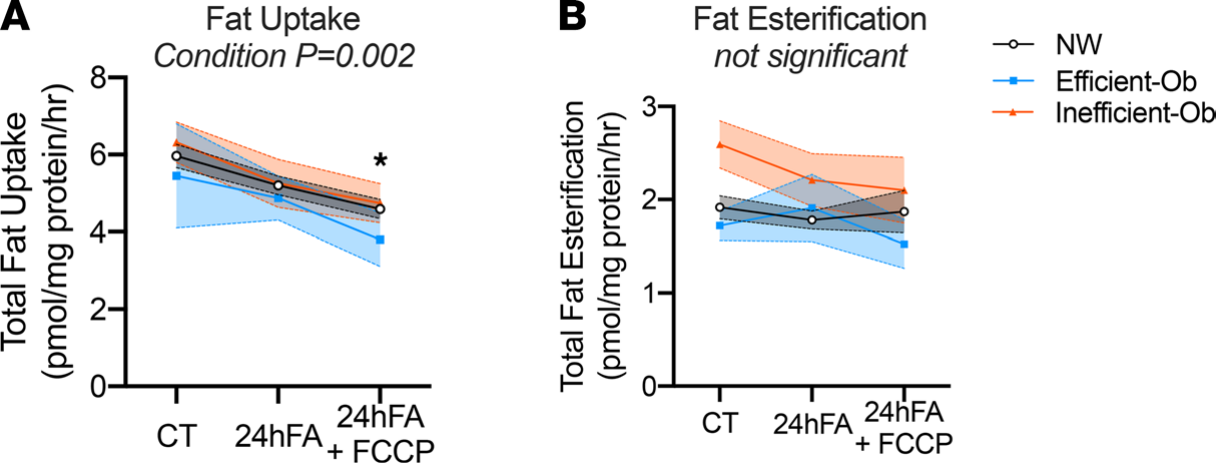
Fat uptake and esterification are not different between MSC metabolic phenotypes. Differences in ASM/CO_2_ among NW, Ef-Ob, and In-Ob MSC metabolism are not explained by differences in fatty acid uptake or esterification. (**A**) Linear fixed effects mixed-model, 2-way ANOVA of fatty acid uptake among MSC metabolic phenotypes (Interaction: *F* = 0.121; *P* = 0.974; Condition: *F* = 7.49, *P* = 0.002; Phenotype: *F* = 0.121; *P* = 0.974). NW *n* = 13; Ef-Ob *n* = 3; In-Ob *n* = 6. Post hoc analysis for Condition revealed that 24hFA+FCCP was lower compared with CT for all groups (*P* = 0.002). (**B**) Linear fixed effects mixed-model, 2-way ANOVA of fat esterification among MSC metabolic phenotypes (Condition: *F* = 0.506, *P* = 0.609; Phenotype: *F* = 2.319; *P* = 0.125; Interaction: *F* = 1.145; *P* = 0.358). NW *n* = 13; Ef-Ob *n* = 3; In-Ob *n* = 6. Data are presented as means ± SEM. *indicates significant difference from CT condition.

We first examined whether fatty acid uptake or esterification rates were different between groups to account for potential differences in cellular fatty acids available for oxidation. We measured total cellular uptake of C^14^-labeled fatty acids and esterification rates in CT, 24hFA, and 24hFA+FCCP conditions. We observed a decrease in total fatty acid uptake during 24hFA+FCCP relative to CT (Figure 2A; *F* = 7.49, *P* *=* 0.002), which was observed in all 3 groups (Figure 2A; *F* = 0.121, *P* = 0.97). This decline in fatty acid uptake with uncoupling plus fatty acid excess may be evidence of a preferential substrate shift from fatty acids to carbohydrate, even in the presence of adequate fatty acid availability. Similarly, fatty acid esterification rates in response to 24hFA or 24hFA+FCCP were not different between NW, Ef-Ob, and In-Ob phenotypes (Figure 2B; *F* = 1.15, *P* = 0.36). These results confirm that cellular fatty acid availability was similar among the groups.

### Mitochondrial lipid availability is not different between MSC metabolic phenotypes

**Figure 3:**
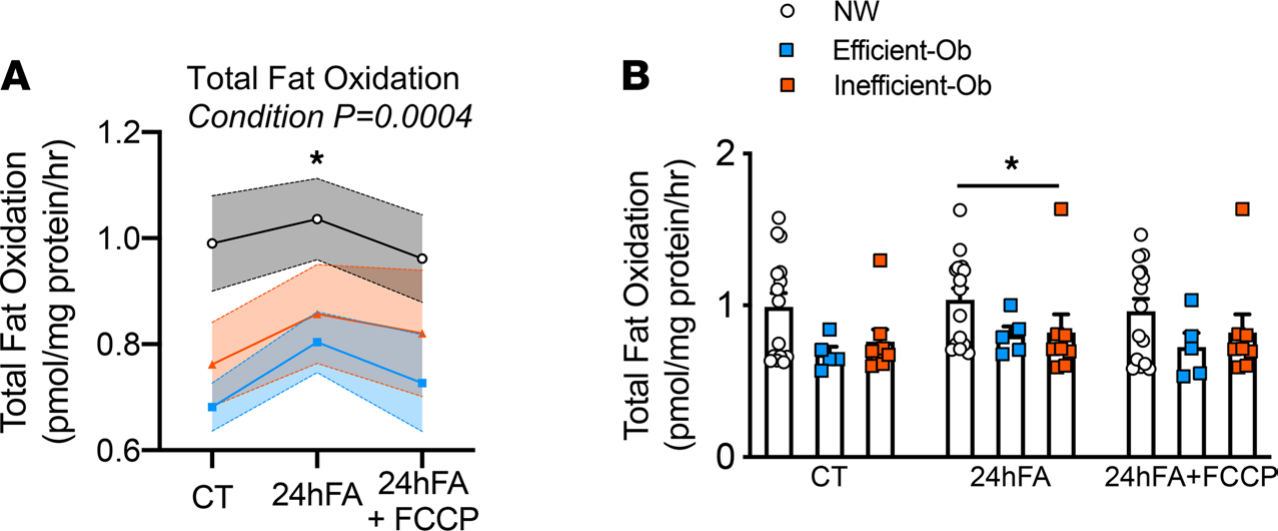
Mitochondrial lipid availability is not different between MSC metabolic phenotypes. Differences in ASM/CO_2_ among NW, Ef-Ob, and In-Ob MSC metabolism are not explained by differences total lipid oxidation. (**A**) Linear fixed effects mixed-model, 2-way ANOVA of total lipid oxidation among MSC metabolic phenotypes (Interaction: *F* = 1.394; *P* = 0.255; Condition: *F* = 9.783, *P* = 0.0004; Phenotype: *F* = 2.060; *P* = 0.149). Post hoc analysis for Condition revealed that the 24hFA condition was elevated compared with both CT and 24hFA+FCCP conditions (*P* = 0.0002 and *P* = 0.02, respectively). NW *n* = 15; Ef-Ob *n* = 5, In-Ob *n* = 8. (**B**) Individual total FAO values shown. Data are presented as means ± SEM. *indicates significant difference from CT and 24hFA+FCCP conditions.

Total fat oxidation is the sum of 2 component measures: complete oxidation of fatty acids to CO_2_ and incomplete oxidation (ASM), which represents fatty acids that entered the mitochondria but were not completely metabolized. Thus, total FAO represents all fatty acids taken up into the mitochondria during the measurement period. As expected, FAO increased from CT with 24hFA and returned to CT levels with 24hFA+FCCP (Figure 3, A and B; *F* = 9.78, *P* = 0.0004); however, these changes were not different between the NW, Ef-Ob, and In-Ob groups. Lactate accumulation rates in response to 24hFA did not change (Supplemental Figure 3, *F* = 0.51, *P* = 0.48) in either MSC group (Supplemental Figure 3, *F* = 0.24, *P* = 0.79), indicating no change in glycolysis. Taken together, these results indicate that, by design, the 24hFA condition increased mitochondrial lipid availability, and this was not different between groups.

### In-Ob MSCs have intrinsic deficits in FAO

The ASM/CO_2_ ratio represents mitochondrial efficiency for FAO, where higher values reflect lower mitochondrial fatty acid flux. This could occur in either β-oxidation or the TCA cycle, given that the complete product is derived from the TCA cycle. ASM/CO_2_ is often higher in skeletal muscle tissue or progenitor cells from animals and humans with established obesity (18, 20–22), and mitochondrial inefficiency for fat oxidation induces insulin resistance in animal models (20).

**Figure 4:**
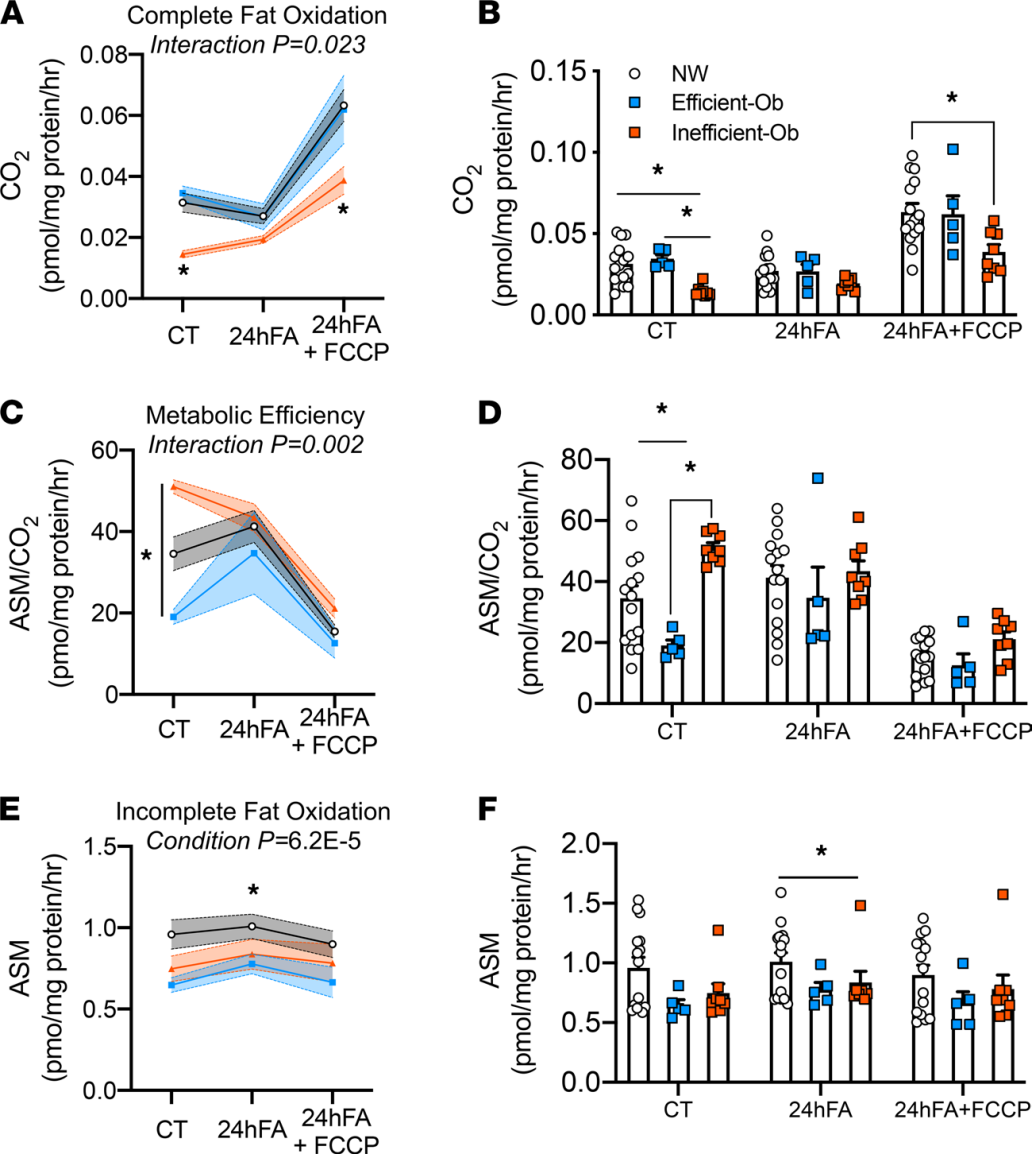
In-Ob MSCs have intrinsic deficits in FAO. (**A**) Linear fixed effects mixed-model, 2-way ANOVA reveals that complete FAO differs among MSC metabolic phenotypes at different experimental conditions, including CT and 24hFA+FCCP (Interaction: *F* = 3.29; *P* = 0.02 indicated by *). NW *n* = 15; Ef-Ob *n* = 5, In-Ob *n* = 8. (**B**) Individual complete FAO values shown. Sidak-adjusted pairwise comparisons reveal increased lipid oxidation in NW versus In-Ob (*P* = 0.002), as well as increased FAO in Eff-Ob versus In-Ob (*P* = 0.006) at CT condition indicated by asterisk. Similarly, NW displays increased complete FAO versus In-Ob at 24hFA+FCCP condition (*P* = 0.017) indicated by asterisk. (**C**) Linear fixed effects mixed-model, 2-way ANOVA reveals 3 distinct MSC metabolic phenotypes assessed by ASM/CO_2_ ratio, an index of metabolic efficiency (Interaction: *F* = 5.319; *P* = 0.002 indicated by *; Condition: *F* = 58.97 *P* = 8.9 × 10^13^; Phenotype: *F* = 4.16; *P* = 0.027). NW *n* = 15; Ef-Ob *n* = 5, In-Ob *n* = 8. (**D**) Individual ASM/CO_2_ values shown. Sidak-adjusted pairwise comparisons reveal increased ASM/CO_2_ in NW versus In-Ob (*P* = 0.024), as well as increased FAO in Ef-Ob versus In-Ob (*P* = 0.001) at CT condition indicated by asterisk. (**E**) Linear fixed effects mixed-model, 2-way ANOVA reveals that distinct MSC metabolic phenotypes are not due to differences in incomplete FAO assessed by ASM (Interaction: *F* = 1.61; *P* = 0.192 indicated by *; Condition: *F* = 12.61; *P* = 6.2 × 10^5^; Phenotype: *F* = 1.96; *P* = 0.161). NW *n* = 15; Ef-Ob *n* = 5, In-Ob *n* = 8. Post hoc analysis for Condition revealed that the 24hFA condition was elevated compared with both CT and 24hFA+FCCP conditions (*P* = 0.0002 and *P* = 0.0005, respectively). (**F**) Individual ASM values shown. Data are presented as means ± SEM.

Mixed-model analysis revealed differences in complete oxidation of fatty acids to CO_2_ among the MSC groups, with a significant Condition by Phenotype interaction (Figure 4A; *F* = 3.29, *P* = 0.02). Post hoc analysis showed complete FAO was lower in In-Ob compared with both NW (Figure 4B, *P* = 0.002) and Ef-Ob (Figure 4B, *P* = 0.006) in the CT condition. This persisted during coincubation with 24hFA+FCCP, where complete oxidation was lower in In-Ob than NW (*P* = 0.02; Figure 4B) and tended to be lower than Ef-Ob (*P* = 0.11; Figure 4B). Though complete oxidation tended to be lower in In-Ob compared with NW in the 24hFA condition, this did not reach statistical significance (*P* = 0.13). We also examined complete oxidation during FCCP incubation alone, which produced similar results as FA+FCCP (Supplemental Figure 4). These results are consistent with increased metabolic rate in the FCCP conditions, where increased metabolism should increase reliance on carbohydrate metabolism. As electron transport and TCA cycle flux increases, acetyl CoA products from β-oxidation will also be metabolized at a faster rate. Thus, we can consider this as a maximal flux of fatty acids through β-oxidation and the TCA cycle.

The ASM/CO_2_ ratio tracked with differences in complete oxidation with a significant Condition by Phenotype interaction (Figure 4C; *F* = 5.32, *P* = 0.002) and higher ratio in In-Ob compared with both NW (Figure 4, C and D; *P* = 0.02) and Ef-Ob (Figure 4, C and D; *P* = 0.0007) in the CT condition. Likewise, in the 24hFA+FCCP condition, ASM/CO_2_ tended to be higher in In-Ob compared with Ef-Ob (Figure 4, C and D; *P* = 0.09). In general, ASM measures tracked closely with total FAO measures, which is expected given that ASM represent the bulk of mitochondrially oxidized fat in a resting cell. ASM increased from CT with 24hFA and returned to CT levels with 24hFA+FCCP (Figure 4, E and F; *F* = 12.60, *P* = 6.15 × 10^5^). These changes were not different between groups.

Together, these metabolic measures highlight several important concepts. First, the experimental conditions achieved changes in fat availability and energetic demand as demonstrated by increased total FAO with 24hFA and increased complete FAO with 24hFA+FCCP, respectively. Second, there were no differences between groups for fat esterification or for cellular uptake or mitochondrial availability of fatty acids. Thus, fat availability does not account for observed differences in complete oxidation. Third, group differences in ASM/CO_2_ ratio were largely driven by differences in complete oxidation of fatty acids to CO_2_, given that ASM measures were not different between groups. Last, increasing mitochondrial lipid availability with 24hFA normalized differences in complete FAO between groups, but the added effect of FCCP demonstrates that, at higher metabolic rates, lipid oxidation through β-oxidation and/or the TCA cycle is impaired in the In-Ob group. Moreover, robust ability to increase FAO in the NW-MSCs indicates that the wide variation in initial metabolic efficiency measures do not necessarily reflect intrinsic capacity for mitochondrial lipid oxidation. Overall, these metabolic flexibility assessments have demonstrated reduced ability to respond to metabolic demand with respect to FAO in the In-Ob group.

### MSC FAO correlates with maternal metabolism

**Figure 5:**
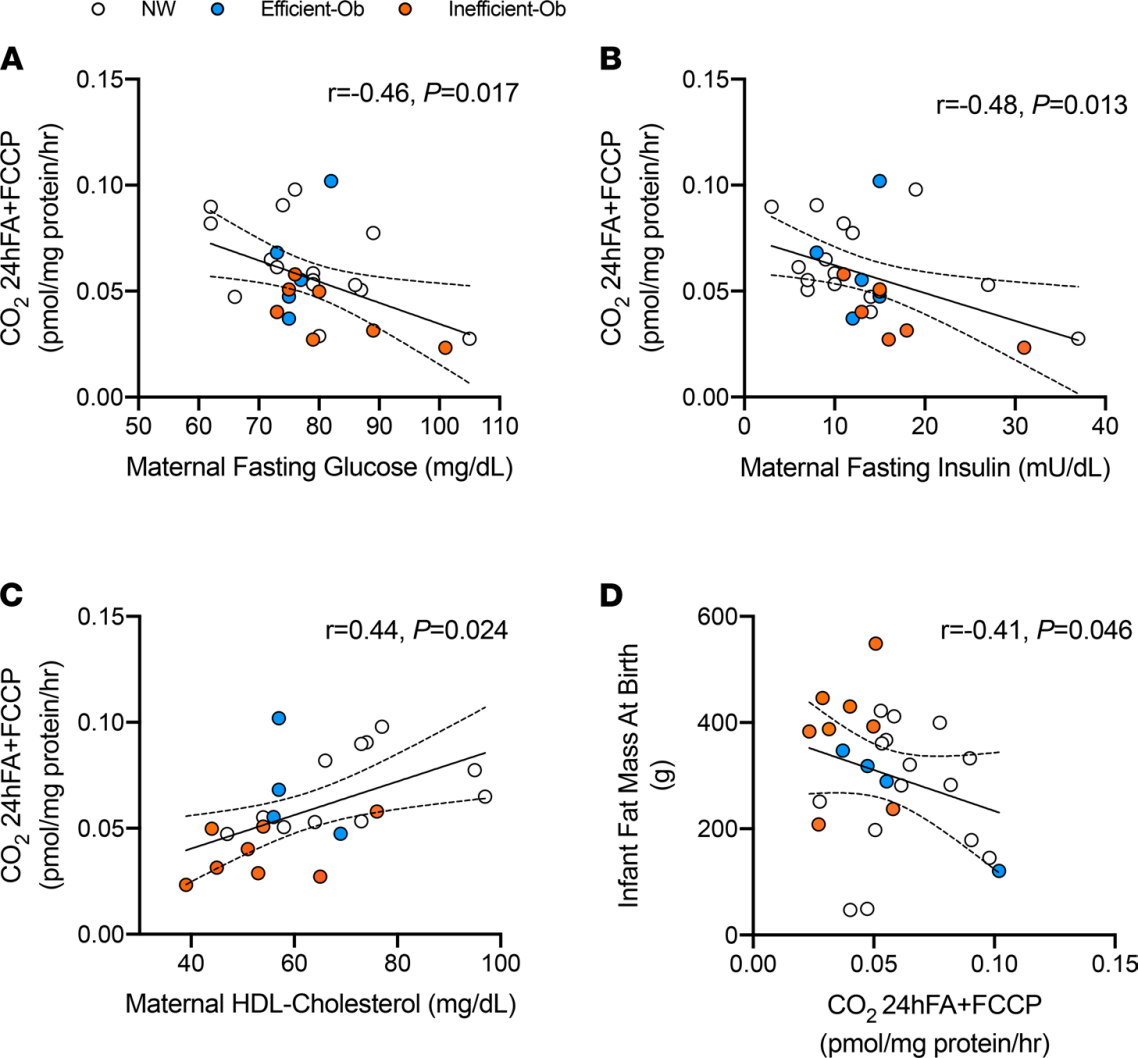
MSC FAO correlates with maternal metabolism and infant fat mass at birth. (**A**) Partial correlation between CO_2_ 24hrFA+FCCP and maternal fasting glucose assessed at late gestation (*r* = –0.46, *P* = 0.017, *n* = 28). (**B**) Partial correlation between CO_2_ 24hrFA+FCCP and maternal fasting insulin assessed at late gestation (*r* = –0.48, *P* = 0.013, *n* = 27). (**C**) Partial correlation between CO_2_ 24hrFA+FCCP and maternal HDL-cholesterol assessed at late gestation (*r* = 0.44, *P* = 0.024, *n* = 23). All maternal correlations were adjusted for maternal BMI and ethnicity. (**D**) Partial correlations between infant fat mass assessed at birth and CO_2_ 24hrFA+FCCP (*r* = –0.41, *P* = 0.046, *n* = 27), adjusted for gestational age, infant sex, and ethnicity. Ninety-five percent confidence intervals shown on all panels.

Despite striking group differences in complete FAO in the 24hFA+FCCP condition, reflecting differences in intrinsic capacity for fat metabolism, we still note a large degree of variation and overlap among individual values across all 3 groups. This overlap among the groups indicates they are not phenotypically distinct and suggests MSC metabolism is likely influenced along a continuum of various in utero exposures, rather than maternal pregravid obesity per se. In our previous work, we observed stepwise changes in maternal metabolic factors for fasting insulin, HOMA-IR, and free fatty acids across MSC groups (14). For the current investigation, we tested correlations among these variables and also maternal fasting total cholesterol, TGs, and glucose (totaling 6 variables) versus MSC FAO after exposure to excess fatty acids and a chemical uncoupler to increase metabolic rate (CO_2_ 24hFA+FCCP). We chose to use this MSC experimental condition because it represents maximal FAO during both metabolic challenges, rather than FAO during basal conditions. Of these tested clinical variables, we observed that fasting maternal glucose (*r* = –0.41, *P* = 0.04; Figure 5A) and fasting insulin (*r* = –0.46, *P* = 0.02; Figure 5B) were negatively correlated with MSC complete FAO, adjusted for maternal BMI and ethnicity. NW-MSCs were interspersed across fasting glucose and insulin correlations, highlighting the potentially influential role of maternal metabolic milieu in MSC metabolism, rather than maternal BMI. In addition, fasting HDL-cholesterol was positively correlated with MSC complete FAO (*r* = 0.45, *P* = 0.02; Figure 5C). Furthermore, infant fat mass assessed at birth was negatively correlated with MSC complete FAO (*r* = –0.41, *P* = 0.046; Figure 5D), adjusted for gestational age, infant sex, and ethnicity.

## Discussion

Maternal obesity alters fetal tissue development in animal models (23–25), providing mechanistic links for intergenerational obesity transmission. In our previous work, we observed that MSCs differentiating into myotubes collected from infants of mothers with obesity were phenotypically different than MSCs from infants of NW mothers, and further examination revealed a bimodal distribution of metabolically efficient and inefficient subtypes among MSCs from infants of mothers with obesity (11). Here, we sought to uncover whether differences in MSC metabolic efficiency reflected a primary defect in FAO by assessing cellular metabolic flexibility. We experimentally challenged cells by altering substrate supply and metabolic rate, which are central components of metabolic flexibility. We found that MSC inefficiency was not due to deficits in substrate supply, as fatty acid uptake and esterification were not different between groups. Furthermore, 24hFA incubation increased lipid entry into the mitochondria (assessed as total FAO), as expected, and this was consistent across all groups, further underscoring that substrate supply does not contribute to the observed metabolic deficits. Rather, the In-Ob MSCs had lower complete fat oxidation compared with both NW and Ef-Ob MSCs (assessed as complete CO_2_) that was not normalized when challenged with a chemical uncoupler or uncoupler plus FA. This indicates an inherent, primary deficit in mitochondrial fat oxidation. Taken together, our series of in vitro experiments revealed that the source of MSC metabolic inefficiency was an inability of the cell to meet the prevailing metabolic demand and thus being metabolically inflexible.

Skeletal muscle plays a primary role in maintaining whole-body metabolic health by regulating substrate metabolism (26). The ability of an organism to modify substrate oxidation during a metabolic challenge, known as metabolic flexibility, is a hallmark of health and can be specific to skeletal muscle (15). We, and others, have previously shown that primary human myotubes cultured from adults with established obesity are unable to robustly increase FAO rates when challenged with excess fatty acids (16–19, 27). To our knowledge, we are the first to report similar findings in MSCs from infants exposed to maternal obesity in utero as early as fetal development. In the current study, a subset of MSCs collected from infants of mothers with obesity were also unable to increase FAO to the same extent as MSCs from NW or Ef-Ob in response to increasing metabolic demand. This limitation in complete fat oxidation is likely due to a primary deficit in the ability to flux lipids through β-oxidation or the TCA cycle. It is possible there may be a secondary deficit in overall cellular respiration or electron transport flux, but this is not likely to be rate limiting for FAO. We also observed reduced TG storage in the In-Ob MSCs compared with Ef-Ob MSCs. We speculate this may be indicative of more favorable lipid storage in Ef-Ob MSCs, as opposed to bioactive lipids such as diacylglycerols or ceramides, perhaps contributing to better metabolic health.

Our findings are consistent with earlier work in which maternal obesity was associated with alterations in fetal skeletal muscle development. In sheep models, fetuses of mothers with obesity have been shown to exhibit defects in skeletal muscle insulin signaling as well as increased fat accumulation and fibrosis (23). Skeletal muscle fiber size may also be detrimentally influenced; maternal obesity has been associated with the downregulation of myogenesis, β-catenin signaling (28), and AMPK signaling (25). In humans, umbilical vein endothelial cells from offspring of mothers with obesity have been shown to exhibit downregulated lipid metabolism and mitochondrial function genes (29). We also observed a downregulation in PI3K and AMPK energy-sensing pathways in MSCs undergoing adipogenesis (30), and in our previous report of myogenic differentiating MSCs, AMPK activation was reduced in In-Ob MSCs compared with NW and Ef-Ob MSCs (11). These earlier reports suggest that maternal obesity may predispose offspring to metabolic disease by altering energy-sensing pathways in myocytes and adipocytes. Here, we are the first to our knowledge to show that maternal obesity is linked to offspring metabolic inflexibility using human cells.

Chronic exposure to excessive maternal substrates in utero increases fetal growth and adiposity (31). For example, elevated glucose that occurs during gestational diabetes is associated with the delivery of infants large for gestational age (32). Similarly, elevated fasting and postprandial TG in maternal obesity predict newborn adiposity (percentage fat) (33). Accordingly, we tested associations between circulating metabolic markers assessed under fasted conditions in late gestation and MSC FAO after excess fatty acid exposure and increased metabolic rate. We observed that FAO (24hFA+FCCP) was inversely correlated with fasting plasma glucose and insulin and positively correlated with maternal HDL-cholesterol, independent of maternal BMI. The overlap of MSC phenotypes within these correlations strengthens the notion that maternal BMI is not the primary determinant of MSC metabolism. The metabolic milieu of the maternal donors of the metabolically inflexible MSCs also tended to have decreased total cholesterol, decreased HDL-cholesterol, and increased HOMA-IR (Table 1). Importantly, these effects were independent of differences in gestational weight gain between groups, and FAO (24hFA+FCCP) was not related to gestational weight gain (data not shown). It may be that chronic maternal substrate exposures are recognized by fetal tissues, including MSCs, as nutrient overload that induces metabolic dysregulation. This may partially explain why lifestyle interventions aimed at reducing gestational weight gain during pregnancy have failed to attenuate infant fat accretion (34–36). Rather, excessive substrate availability in the in utero metabolic milieu may contribute to altered MSC metabolic flexibility. Epigenetic mechanisms by which intrauterine exposures alter fetal tissue development are well established in animal models (37, 38), though these mechanisms are difficult to define in humans. We previously reported that MSCs from infants of mothers with obesity have hypermethylation and lower mRNA content of genes regulating FAO and AMPK activity (11). We postulate that maternal substrates, which are typically elevated with maternal obesity, may alter MSC epigenetic signatures and subsequent metabolic function. These associations provoke the idea that normalization of the metabolic milieu in women with pregestational obesity may reprogram MSC metabolism, including metabolic flexibility, and improve offspring outcomes.

Consistent with maternal metabolic milieu, we observed differences in infant body composition between MSC metabolic phenotypes and also a linear decrease in fat-free mass and linear increase in fat mass and cord blood insulin across the MSC metabolic phenotypes (NW, Ef-Ob, and In-Ob, respectively). We observed that MSC FAO (24hFA+FCCP) was inversely correlated with neonatal fat mass after adjusting for gestational age, infant sex, and ethnicity. This correlation suggests that greater infant fat mass is related to reduced metabolic health of myogenic differentiating MSCs, which are the precursors to skeletal muscle tissue. These results are similar to findings from animal models, which show that maternal obesity is linked to greater lipid deposition in skeletal muscle, smaller muscle fibers (28), and reduced oxidative metabolism in muscle-derived progenitor cells from offspring skeletal muscle tissue (39). Overall, these potentially novel observations support the notion that maternal metabolic health contributes to altered MSC oxidative metabolism, which may play a role in fetal fat accrual, as suggested by the correlation with neonatal fat mass. However, it must be noted that none of the mothers had developed gestational diabetes, or even clinically elevated fasting glucose or insulin levels, nor were any of the infants born large for gestational age. Our observations are distinguishing metabolic and phenotypic differences among a relatively healthy group of mothers and infants. Therefore, whether this infant phenotype drives body composition and skeletal muscle metabolism during childhood is an ongoing investigation in the Healthy Start Study cohort.

Here, we have demonstrated that a subset of MSCs from infants born to mothers with obesity had lower complete fat oxidation compared with both NW and Ef-Ob MSCs that did not normalize when metabolically challenged with a chemical uncoupler or uncoupler plus excess fatty acids. While the causes of intrinsic deficits in FAO are not fully understood, this work shows that it may be evident as early as fetal development, and our previous report indicates it may be epigenetically programmed in the MSCs (11). Associations between maternal metabolic health markers and MSC FAO suggest that MSC metabolic programming is driven in part by the maternal metabolic milieu during gestation. The observed associations between infant fat mass and MSC FAO indicate that a less favorable maternal metabolic milieu may induce a primary deficit in skeletal muscle FAO during fetal development, and this is related to greater adiposity as early as birth. Whether clinical intervention restores maternal metabolism and improves fetal tissue development may be of paramount importance for improving body composition and slowing the development of metabolic syndrome in adolescents. Future studies should address this link, as these findings have the potential to impact clinical practice by providing identifying modifiable targets that promote metabolic health at birth and during childhood.

## Methods

### Participants

As described previously, MSCs were collected from 165 infants born to mothers participating in the Healthy Start Study (12). Briefly, women were eligible for enrollment if they were 16 years of age or older, currently pregnant, and carrying a singleton at least 23 weeks of gestation. Women were excluded for prior diabetes, prior premature birth, serious psychiatric illness, or multiple pregnancies.

#### Maternal phenotyping.

Maternal metabolic phenotyping from the Healthy Start Study has been published elsewhere (7). As described, women were characterized at midgestation (median of 17 weeks). Assessments included demographics, tobacco use, height, and weight. Pre-pregnancy BMI was obtained through medical records (84%) or self-report (16%). Fasting blood samples collected at midgestation were analyzed for glucose, insulin, TG, and free fatty acids.

#### Infant phenotyping.

Infant weight at time of birth was obtained through medical records. Additionally, infant weight, length, and body composition were assessed within 24–48 hours after birth using whole-body air plethysmography (PEA POD, COSMED, Inc). Body composition consists of estimated fat mass and fat-free mass.

### MSC procedures

The MSC culture and isolation procedures have been described previously (12). In brief, MSCs were cultured from umbilical cord explants, and all experiments were conducted on cells within passages 4–6. Myotubes were differentiated for 21 days using myogenic induction media (produced in-house; ref. 12) and then collected for protein or fat content measures or exposed to experimental conditions for FAO assays.

### Measures of stored fats

Following 21 days of myogenesis, cells were fixed with 4% formaldehyde and stained for ORO lipid content, as described (12). For TG measures, following 21 days of myogenesis, cells were harvested in ice-cold PBS, then pelleted by centrifugation for 5 minutes at 1000*g* at 4°C and flash frozen in liquid nitrogen. Cells were thawed and resuspended and lysed in PBS. An aliquot was removed for measures of total protein content by bicinchoninic acid (BCA) assay (Thermo Fisher Scientific). Then, TG content was measured as described by us (40) using a modified Bligh and Dyer method (41). Resultant TG concentrations were normalized to starting protein concentration.

### Protein content

Cells were harvested in lysis buffer (CelLytic MT, MilliporeSigma) supplemented with protease and phosphatase inhibitor cocktails (MilliporeSigma). Total protein was determined by BCA assay. Protein content of PPARγ, SREBP1, FAS, SCD1, DGAT1, and ACS, with β-actin as reference control was determined by Simple Western size-based protein assay (Wes, ProteinSimple) following manufacturer’s protocol and as described (11). Results from Wes were analyzed using ProteinSimple Compass software. All antibodies were optimized in-house for this system, and antibody specifics and assay conditions are listed in Supplemental Table 1. Antibodies and conditions are detailed in Supplemental Table 1.

### ^14^C-FAO metabolism assays

^14^C-labeled FAO was assessed as previously described (14). Briefly, 21-day myogenic differentiating MSCs were incubated with regular myogenic media containing 200 μM oleate and palmitate (2:1 ratio) with 1 mM carnitine, spiked with 0.25 μCi/mL [^14^C]-oleate and 0.25 μCi/mL [^14^C]-palmitate (PerkinElmer Life Sciences), as described (11) After a 2-hour incubation period, the rate of FAO was determined by measuring ^14^CO_2_ released from the media after acidification with perchloric acid. All measurements were performed in triplicate. This method allows for assessment of both complete FAO to CO_2_ as well as incomplete FAO, quantified as ASM. The sum of CO_2_ and ASM measures represents total FAO and the amount of ^14^C fatty acids that entered the mitochondria. In addition, fatty acid esterification was measured using ^14^C-esterified fatty acids extracted with chloroform/methanol. Total cellular fatty acid uptake was calculated as the sum of ^14^C measured in the cell lysate plus total FAO. These measures were made in 3 conditions following 21 days of myogenesis: (a) myogenesis alone (CT), (b) following 24 hours of excess fatty acid exposure (24hFA; 200 μM oleate and palmitate [2:1 ratio] plus 1 mM carnitine), and (c) following 24hFA, with FAO measures in the presence of 8 μM FCCP, a chemical uncoupler (24hFA+FCCP).

### l-Lactate index of glycolysis

Following 21 days of myogenic induction, spent media were replaced with fresh media. Cells were incubated 2 hours at 37°C, and then l-lactate accumulation was measured in the media using the Glycolysis Cell-Based Assay Kit (Cayman Chemical). Data were corrected for values in a no-cell control well and for total protein content, as measured by BCA method.

### Statistics

Data are presented as mean ± SEM unless otherwise noted. Homogeneity of variances was tested using Levene’s test. For comparison of participant characteristics listed in Table 1, linear 1-way ANOVA was used. For comparisons of 3 groups for lipids and protein measures, data were analyzed using 2-way ANOVA with planned contrasts to evaluate the data as 3 independent groups, as well as NW versus both Ef-Ob and In-Ob groups. This was to account for the possibility that some variables would be similar between Ef-Ob and In-Ob groups, while testing for individual-group differences. All ANOVAs were considered significant at *P* < 0.05. For experimental conditions, fixed effects linear mixed models were computed in SPSS to test for an interaction effect of group by condition (repeated measure). If no interaction was observed, the main effects of group and condition were interpreted. Post hoc pairwise testing was adjusted for multiple comparisons using Sidak’s adjustments.

Correlation analyses were used to test associations between FAO assays and maternal metabolism during midgestation, computed in GraphPad Prism. Data were tested for normality using D’Agostino’s and Pearson’s tests. Partial correlations were used to determine relationships between MSC metabolic phenotype and maternal or infant variables. Maternal correlations were adjusted for maternal BMI and self-reported ethnicity (42). Infant correlations were adjusted for gestational age, infant sex, and maternal ethnicity. Based on a relatively small number of tested variables, we did not adjust for multiple comparisons.

### Study approval

Umbilical cord tissue samples used in this study were originally collected from participants volunteering for the Healthy Start Study (Clinical Trials.gov, NCT02273297). This study was approved by the Colorado Multiple Institutional Review Board, Aurora, Colorado, USA. Written informed consent was obtained from all study participants prior to study inclusion.

## Author contributions

MLE analyzed and interpreted the data and drafted the manuscript. ZWP conceptualized the study, conducted experiments, acquired data, and edited and approved the manuscript. AMD conducted experiments, acquired data, and edited and approved the manuscript. DD conceptualized and implemented the parent Healthy Start Study and edited and approved the manuscript. LMR analyzed and interpreted data and edited and approved the manuscript. KEB conceptualized the study, conducted experiments, acquired data, analyzed and interpreted data, as well as edited and approved the manuscript.

## Supplementary Material

Supplemental data

## Figures and Tables

**Table 1:**
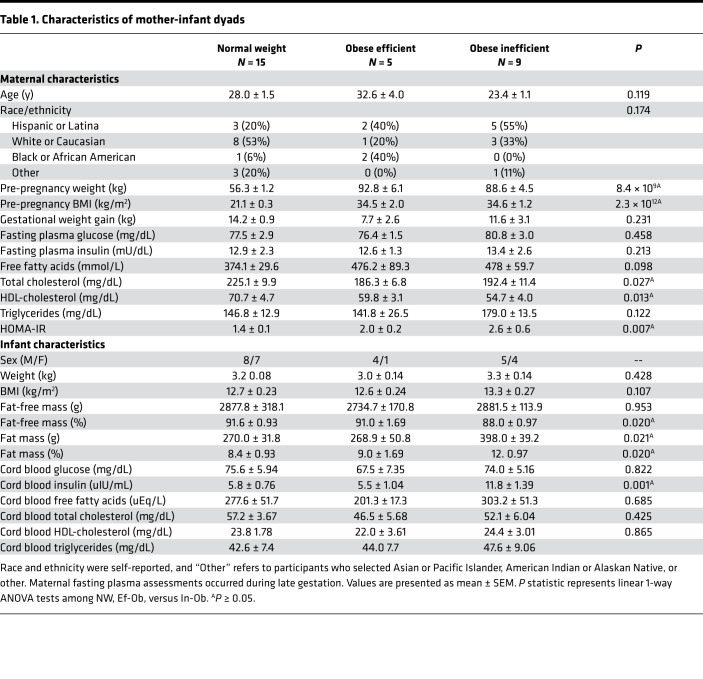
Characteristics of mother-infant dyads

## Version 1 06/01/2021

In-Press Preview

## Version 2 07/08/2021

Electronic publication
