# Supplementary material for: Maternal metabolic health drives mesenchymal stem cell metabolism and infant fat mass at birth
Source: JCI Insight. 2021 Jul 8;6(13):e146606. doi: 10.1172/jci.insight.146606 (PMC8410068; doi:10.1172/jci.insight.146606)
Supplement: Supplemental data [file jciinsight-6-146606-s043.pdf]

Supplemental Table 1. ProteinSimple WES Antibody and Assay Conditions

Antibody	Manufacturer	Cat. No.	Ab Dilution	Sample [protein]
β-Actin	Cell Signaling Technology	4970	1:50	0.2 µg/µL
PPARγ	Cell Signaling Technology	2435	1:25	0.8 µg/µL
SREBP1	Novus Biologicals	NB100-2215	1:25	0.2 µg/µL
FAS	Cell Signaling Technology	3180	1:500	0.2 µg/µL
SCD1	R&D Systems	AF7550	10 µg/mL	0.2 µg/µL
DGAT1	Novus Biologicals	NB100-57086	1:50	0.4 µg/µL
ACS	Cell Signaling Technology	3658	1:200	0.2 µg/µL

SUPPLEMENTARY FIGURE 1:

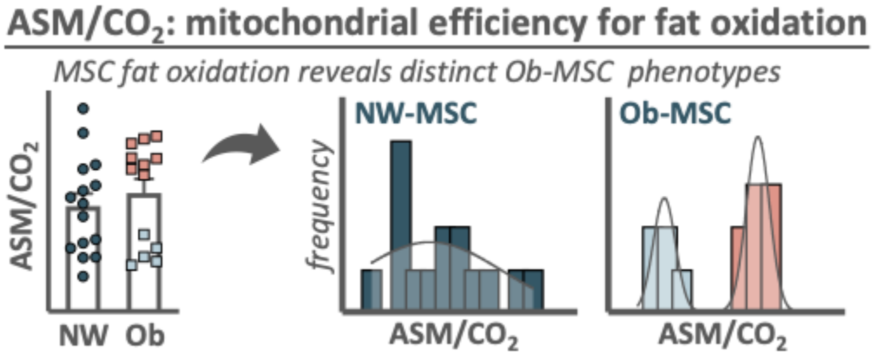

Previously reported MSC metabolism distinguishing Ob-MSC groups (11). Reprinted from *Molecular Metabolism*, 6(11), Kristen E. Boyle, Zachary W. Patinkin, Allison L.B. Shapiro, Carly Bader, Lauren Vanderlinden, Katerina Kechris, Rachel C. Janssen, Rebecca J. Ford, Brennan K. Smith, Gregory R. Steinberg, Elizabeth J. Davidson, Ivana V. Yang, Dana Dabelea, and Jacob E. Friedman, Maternal obesity alters fatty acid oxidation, AMPK activity, and associated DNA methylation in mesenchymal stem cells from human infants, 1503–1516, Copyright (2017), with permission from Elsevier.

SUPPLEMENTARY FIGURE 2: ProteinSimple WES chemiluminescent protein measures

S2A. PPARγ

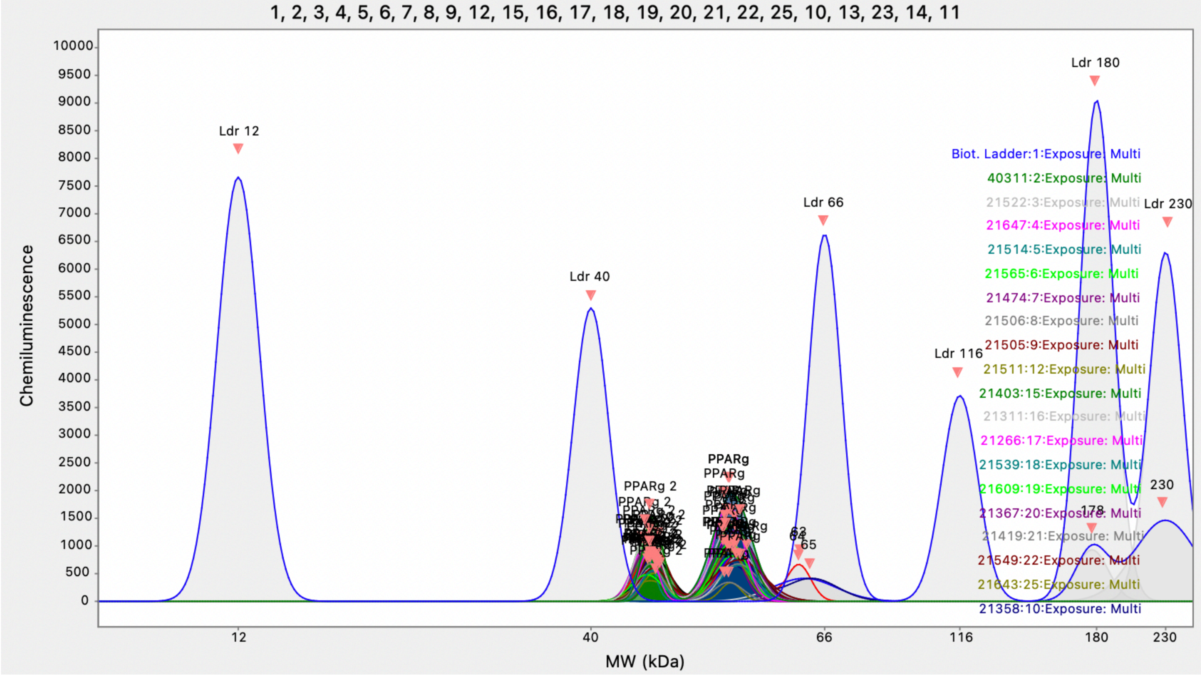

S2B. SREBP1

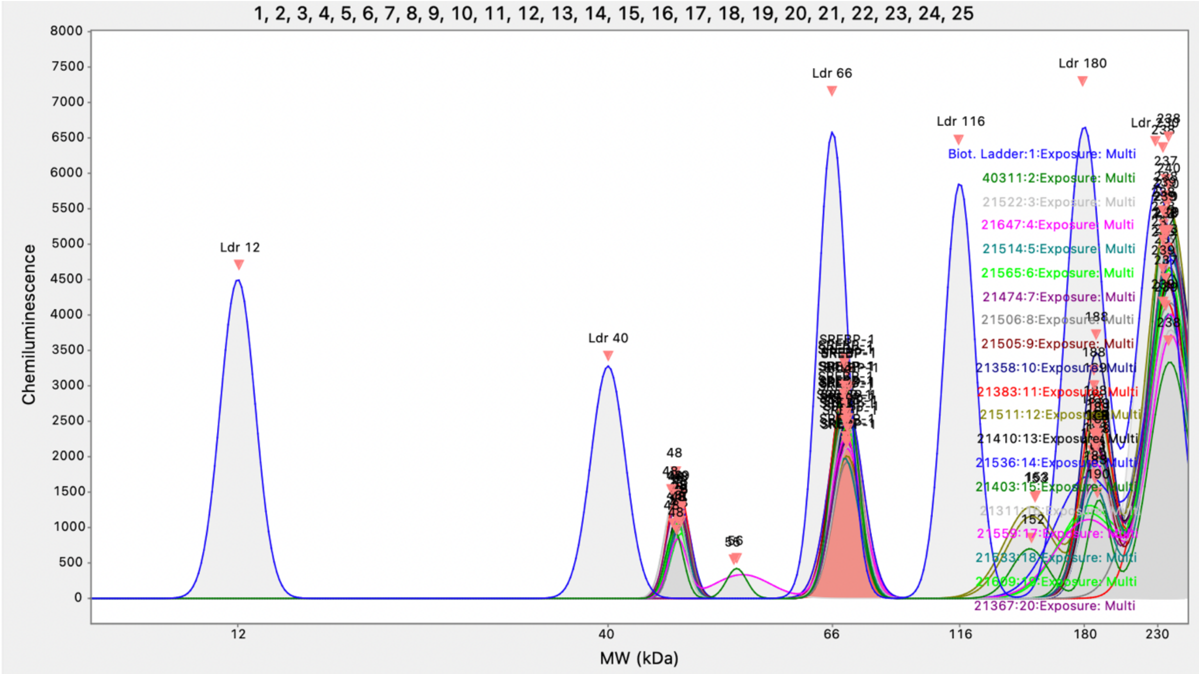

S2C. FAS

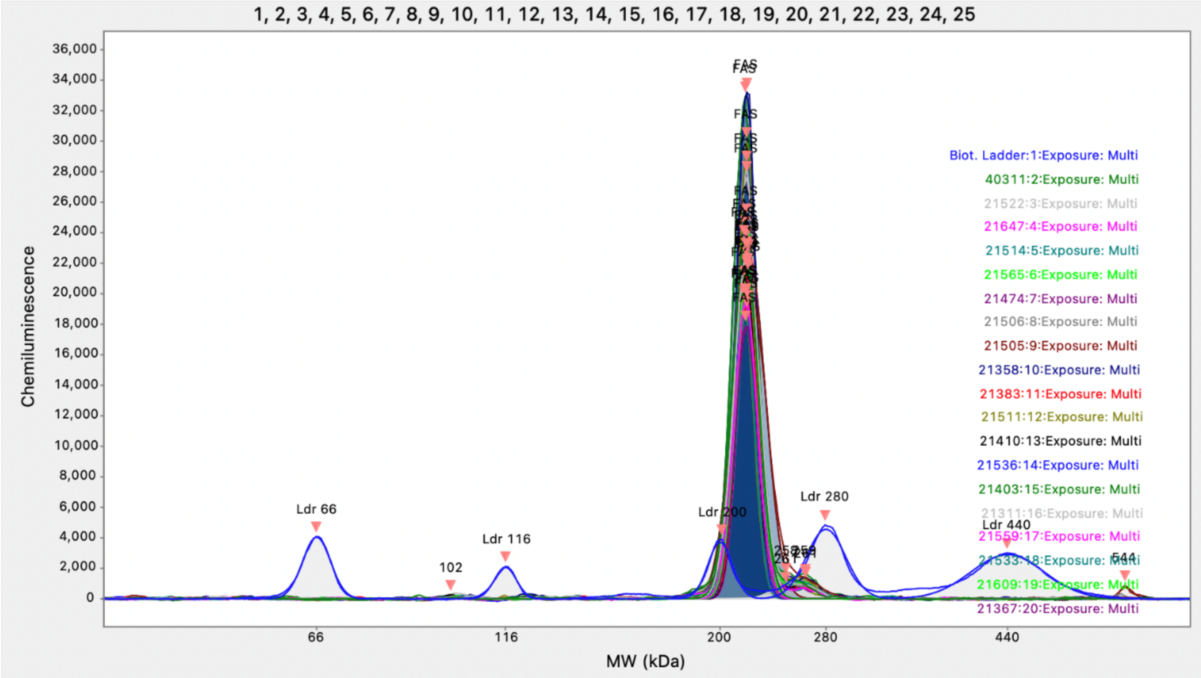

S2D. SCD-1

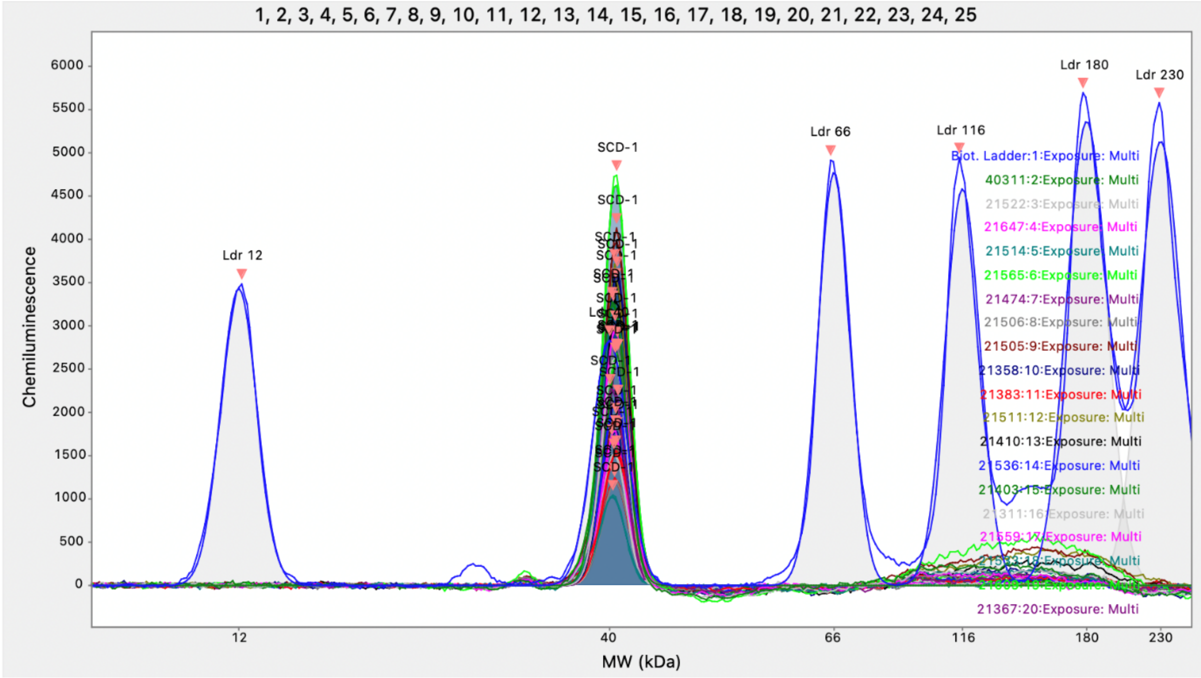

S2E. DGAT

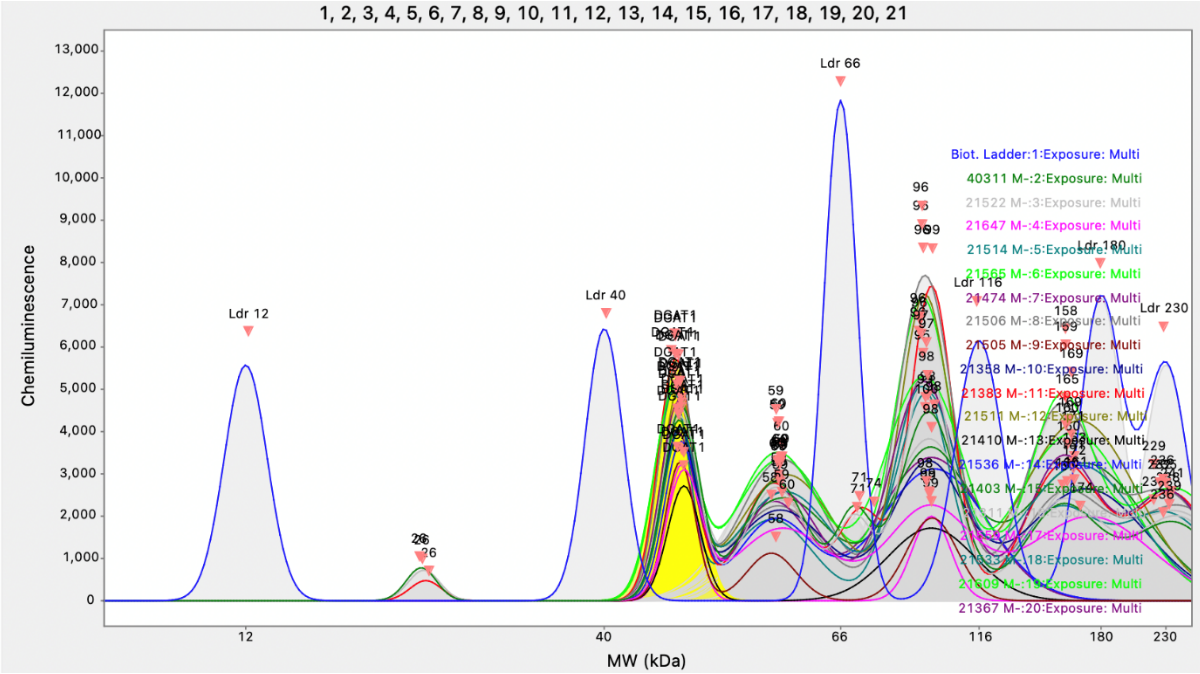

S2F. ACS

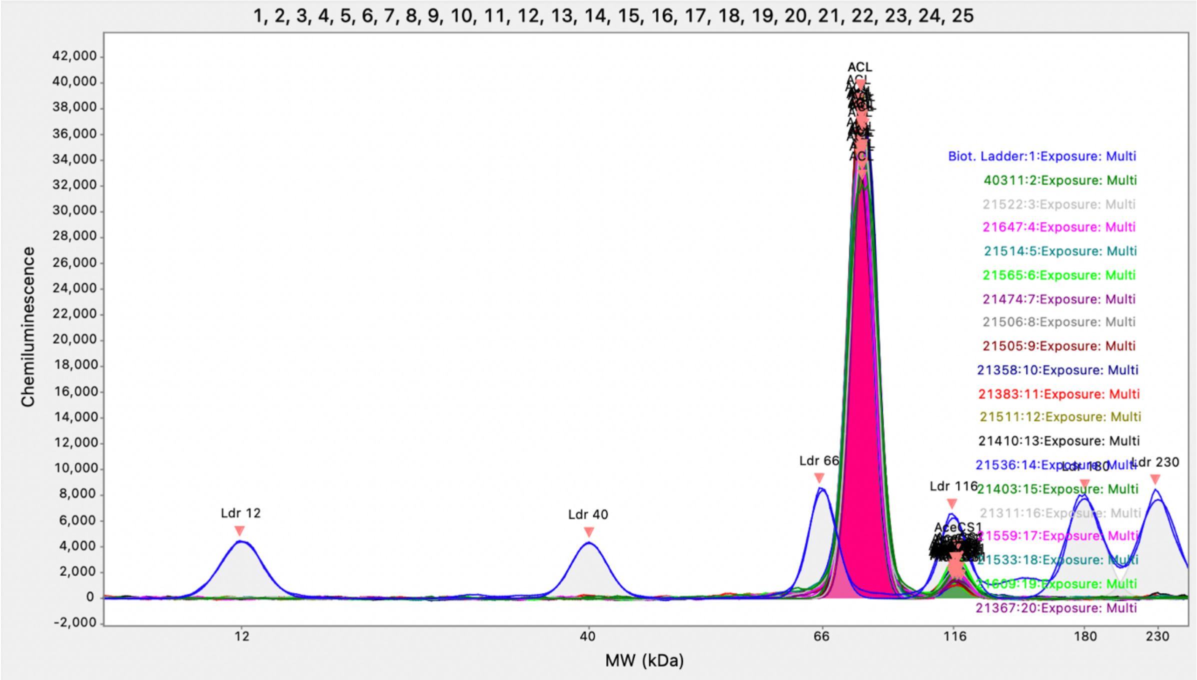

***Please note ACS (at MW ~118 kDa) was run multi-plexed with an additional protein at 81 KDa.*

S2G. β-actin

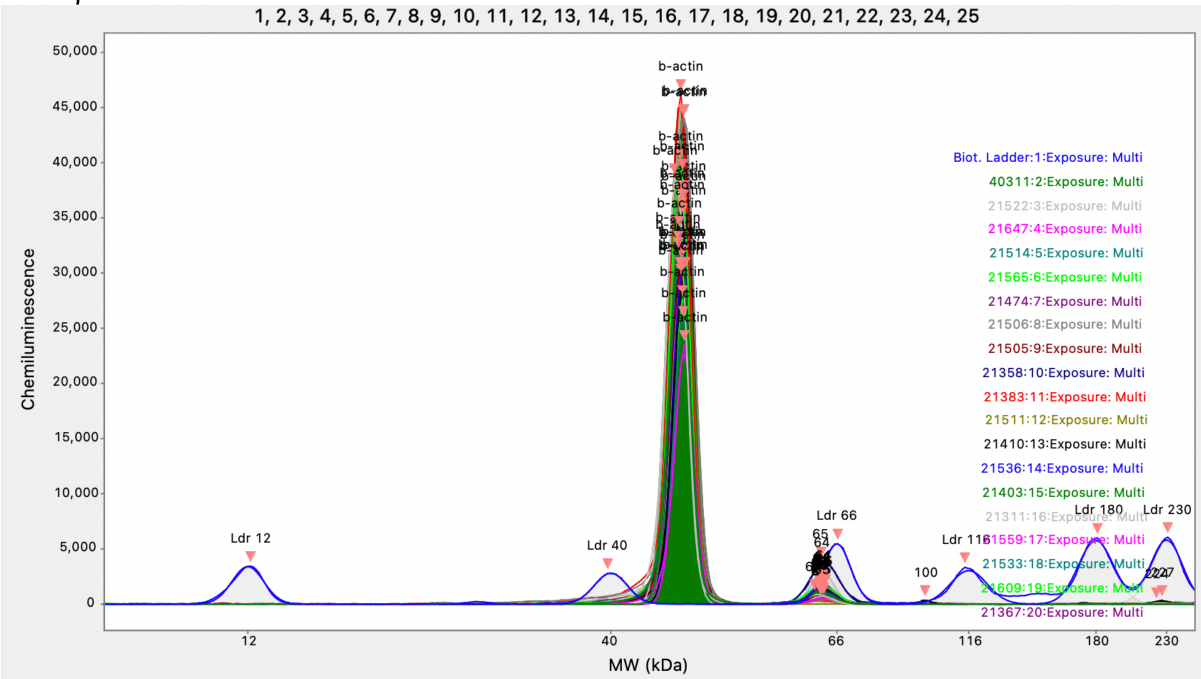

1, 2, 3, 4, 5, 6, 7, 8, 9, 10, 11, 12, 13, 14, 15, 16, 17, 18, 19, 20, 21, 22, 23, 24, 25
Chemiluminescence
50,000
45,000
40,000
35,000
30,000
25,000
20,000
15,000
10,000
5,000
0
b-actin
Ldr 12
Ldr 40
Ldr 66
Ldr 116
Ldr 180
Ldr 230
100
Biot. Ladder:1:Exposure: Multi
40311:2:Exposure: Multi
21522:3:Exposure: Multi
21647:4:Exposure: Multi
21514:5:Exposure: Multi
21565:6:Exposure: Multi
21474:7:Exposure: Multi
21506:8:Exposure: Multi
21505:9:Exposure: Multi
21358:10:Exposure: Multi
21383:11:Exposure: Multi
21511:12:Exposure: Multi
21410:13:Exposure: Multi
21536:14:Exposure: Multi
21403:15:Exposure: Multi
21367:20:Exposure: Multi
12
40
66
116
180
230
MW (kDa)

SUPPLEMENTARY FIGURE 3:

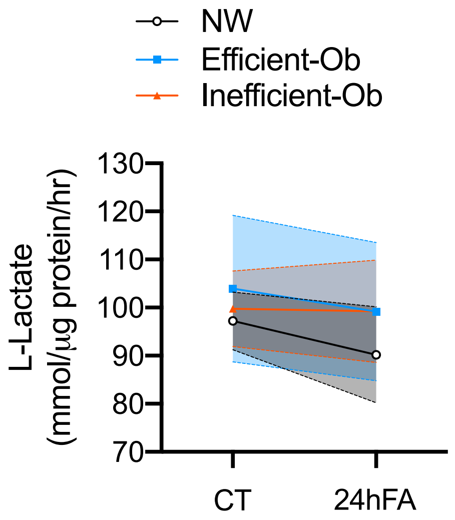

Lactate concentrations in media, an index of glycolysis, were not different across NW, Ef-Ob, and In-Ob MSCs. Mixed model analysis of glycolysis among MSC metabolic phenotypes during two experimental conditions: control and 24hFA exposure (Interaction: F=0.116; *P*=0.891; Condition: F=0.510, *P*=0.483; Phenotype: F=0.238; *P*=0.790).

SUPPLEMENTARY FIGURE 4:

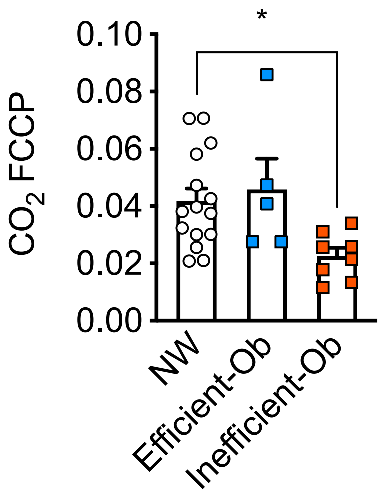

One-way ANOVA indicates significant between group effects (F=4.486, P=0.022). Sidak-adjusted post-hoc comparisons reveal Inefficient-Ob is significantly reduced compared to normal weight (P=0.038) indicated by a and b symbols. Data are presented as means ± SE. * indicates difference between NW and In-Ob ($P \leq 0.05$).
